# Effect of Crushed Flaxseed Consumption on Cardiovascular Risk Indicators in Menopausal Women

**DOI:** 10.3390/life14070849

**Published:** 2024-07-05

**Authors:** Petra Lenártová, Martina Gažarová, Jana Kopčeková, Jana Mrázová

**Affiliations:** Institute of Nutrition and Genomics, Faculty of Agrobiology and Food Resources, Slovak University of Agriculture in Nitra, Trieda Andreja Hlinku 2, 949 76 Nitra, Slovakia; martina.gazarova@uniag.sk (M.G.); jana.kopcekova@uniag.sk (J.K.); jana.mrazova@uniag.sk (J.M.)

**Keywords:** flaxseed, lipid profile, visceral fat area, hs-CRP, menopausal women

## Abstract

Flaxseed is known for its numerous health benefits and is often used in the prevention and treatment of civilizational diseases. This study aimed to evaluate the impact of consuming crushed flaxseed on cardiovascular risk in 51 menopausal women. The intervention lasted for 8 weeks, during which participants received a daily dose of 40 g of crushed flaxseed from two varieties with differing lignan contents. Participants were divided into three subgroups based on the variety of flaxseed consumed: (1) high-lignan group (HL), (2) low-lignan group (LL), and (3) control group (no flaxseed consumption). Biochemical blood parameters were measured using a BiOLis 24i Premium automatic analyzer. Body composition was assessed using an InBody 720 device. In the lipid profile, we observed a significant increase in total cholesterol (T-C) and high-density lipoprotein cholesterol (HDL-C) levels, along with a significant decrease in low-density lipoprotein cholesterol (LDL-C) levels in both the HL and LL groups. Triglyceride (TG) levels exhibited no significant change, whereas high-sensitivity C-reactive protein (hs-CRP) levels were significantly reduced in both the HL (*p* < 0.01) and LL (*p* < 0.01) groups. Visceral fat area (VFA) and percent body fat (PBF) showed a slight decreasing trend in the HL group, whereas in the LL group, VFA showed a slight increase. Body mass index (BMI) remained stable across all groups. These findings suggest that for the modulation of cardiovascular disease (CVD) risk factors, the daily dosage, duration of consumption, form of intake, and the specific variety of flaxseed (based on lignan content) are crucial factors.

## 1. Introduction

Flaxseed is a unique source of nutrients with a wide range of beneficial properties for the human body. It is used not only in the prevention of many civilizational diseases but also in their treatment [1,2].

Flaxseed is rich in alpha-linolenic acid, fiber, lignans, and alpha-tocopherol (vitamin E), making it an excellent source of oil, fiber, lignans, and other substances in nutrition. The therapeutic properties of flaxseed are employed in the treatment of diabetes, hypercholesterolemia, atherosclerosis, and disorders of fat metabolism. Additionally, it has antiatherogenic, antioxidant, and anti-inflammatory properties [2,3,4,5,6].

Several randomized studies have reported the antihypertensive effects of flaxseed [7]. These effects have been attributed to the ability of flaxseed to change the pro-inflammatory effect of oxylipin concentration, reduce the production of inflammatory cytokines [8] and inhibit prostacyclin, improve heart rhythm and positively influence cell apoptosis [9].

In addition to the previously mentioned benefits, flaxseed consumption may also prevent and control a number of other conditions, including neurodegenerative disorders, obesity, polycystic ovary syndrome, gout, liver and kidney dysfunction, postmenopausal symptoms, osteoporosis, irritable bowel syndrome, dry eye disease, cystic fibrosis, diarrhea, and cancer, particularly of the mammary and prostate glands [10,11].

Secoisolariciresinol diglucoside (SDG) is an important substance for the body as it has many positive biological effects and health benefits [12]. For example, secoisolariciresinol diglucoside (SDG) has antioxidant and antisclerotic properties. SDG is metabolized in the digestive tract to the compound secoisolariciresinol (SECO), which is subsequently converted by the intestinal microflora to the mammalian lignans enterodiol (ED) and enterolactone (EL). EL and ED protect somatic cells from DNA damage and lipid peroxidation. The similarity of EL and ED to estradiol allows these lignans to bind to estrogen receptors and exert weak estrogenic or antiestrogenic effects. This mechanism makes it possible to relieve typical menopausal symptoms in premenopausal and menopausal women [13,14,15].

An interesting finding from a more detailed analysis of flax varieties was that the varieties with a brown seed color had the highest average values of saturated fatty acids, proteins, fiber, tocopherol, phenolics, SDG, and SECO lignans. Varieties with a yellow seed color were characterized by a higher content of unsaturated fatty acids and mucilage. These differences can be used in nutritional therapy in accordance with the desired effect on health and metabolism [16]. Flaxseed can significantly reduce the intensity of symptoms associated with menopause, constipation, and mental fatigue. It also improves skin condition and speeds up wound healing. In addition to bioactive compounds, flaxseed contains antinutrients such as cyanogenic glycosides (CGs), trypsin inhibitors, and phytic acid, which can reduce the bioavailability of essential nutrients and/or limit their health benefits [17,18]. The form of flaxseed consumed also affects the bioavailability of alpha-linolenic acid (ALA) and lignans. To ensure a high level of bioavailability of its bioactive compounds, flaxseed should be consumed in ground form [17]. The stability of flaxseed and its bioactive compounds depends on the form consumed. Grinding flaxseed breaks the barrier of the seed coat and increases its susceptibility to oxidation. Therefore, flax must be stored under appropriate conditions (airtight container and appropriate temperature and humidity) [19]. In summary, ground flaxseed should be used as soon as possible after grinding and stored for a short time. On the other hand, whole flaxseed provides the lowest amount of ALA and SDG. This is because it is covered by a hard and impermeable seed coat [20,21]. In order to adjust the lipid profile and control CVD, it is necessary to consume 10–60 g of flaxseed per day on a regular basis [22].

Other factors that influence the health effects of flax include genetics, climate, and growing conditions. For instance, seeds grown in warmer conditions were more effective and had more chemical components [16]. Significant variations in fatty acid composition, phenolic acids, and lignans were observed in flaxseed varieties from different countries. Therefore, the cultivar and the origin of the seed significantly influence the nutritional composition (fatty acids, phenolic acids, and lignans) and subsequent antioxidant activity of the flaxseed [23].

The objective of this study was to assess the effect of ground flaxseed consumption on cardiovascular risk in a sample of 51 menopausal women. 

## 2. Materials and Methods

### 2.1. Probands

The monitored group comprised 51 menopausal women from the general population, selected at random within the age range of 45–55 years. The monitored group was divided into three monitored groups, each comprising 17 probands. The groups were as follows: the first group consumed high-lignan flaxseeds of the Agram variety (HL), the second group consumed low-lignan flaxseeds of the Raciol variety (LL), and the third was a control group without flaxseed consumption. The following criteria were established to guide the inclusion or exclusion of probands. Inclusion criteria for the study included female gender, age (45–55 years), menopause, good health, the absence of serious acute or chronic diseases, the absence of food allergies and intolerances, and the absence of drug treatment, as these factors could affect the results of the clinical study. Additionally, participants were required to maintain their dietary habits, diet, physical activity, and lifestyle throughout the study period. The study excluded individuals with serious diseases, undergoing medical treatment, or following a special dietary regimen recommended by a physician. Furthermore, the study excluded individuals who had undergone implantation of an endoprosthetic device or who were wearing a pacemaker. The condition of participation in the study was the submission of information about the nature, course, and conditions of the study in oral and written form, which all enrolled probands confirmed by signing the informed consent. All female participants were recruited to this study on a voluntary basis. They were not subjected to any form of coercion or offered any form of financial or other inducement to participate in the study.

### 2.2. Intervention

The intervention lasted eight weeks and comprised a daily ration of 40 g of crushed flaxseeds of two varieties, differing in their lignan content. Two varieties of flaxseed were included in the study: Agram and Raciol. Agram is distinguished by a higher content of lignans (6248.0 mg/kg SECO brown seed color) and Raciol by a lower content of lignans (4297.2 mg/kg SECO; yellow seed color). Both varieties were supplied by AGRITEC Co., Ltd., Šumperk, Czech Republic. The daily ration of flax (freshly crushed flaxseeds) was packaged in special food bags with a sealable opening. The bags were constructed from a material that does not permit light transmission, with a protective barrier against oxidation. The daily rations of flaxseeds were crushed and distributed to all subjects on a weekly basis. It was recommended that the subjects store the bags of crushed flaxseeds in the refrigerator.

A standardized procedure was employed to analyze the nutritional composition of the flaxseeds. This procedure was initiated prior to the commencement of the intervention. The determination of the SECO content was conducted on homogenized flaxseeds following the previous extraction of fat in a Soxhlet extractor. The method is based on acid hydrolysis of secoisolariciresinol glycosides (SDG and oligomeric forms of bound lignan) by extraction of aglycones with organic solvents and subsequent determination of derivatized SECO by gas chromatography with flame-ionization detection after the addition of an internal standard (nordihydroguaiaretic acid). The determination of fatty acids was carried out according to ČSN ISO 5508 [24] i.e., the technical standard for the analysis of fatty acid methyl esters by gas chromatography using the software Clarity chromatography version number 9.1.01.013 (DataApex Co., Ltd., Prague, Czech Republic). Table 1 shows the characteristics of selected nutritional values of the consumed flaxseed varieties.

### 2.3. Characteristics of the Used Methods and Study Design

#### 2.3.1. Determination of Biochemical Blood Parameters

Prior to and following the eight-week intervention, blood samples and anthropometric measurements were obtained from subjects with a pre-established flaxseed genotype. Blood samples were collected following a 10–12-h fasting period, in accordance with standard procedures. A qualified medical professional punctured a peripheral vein in the elbow of each seated subject using a sterile needle and collected the blood into a dry tube. Blood samples were collected from each subject and placed into a 7.5-mL serum gel tube. The samples were centrifuged at 3000 rpm for 10 min at 4 °C, and the serum was used for analysis. Blood biochemical parameters were detected using the Automated Clinical Analyzer BiOLis 24i Premium (Tokyo Boeki Medical System Co., Ltd., Tokyo, Japan). This device employs end-point photometry (turbidimetry), kinetics, and homogeneous immunoanalysis. The following parameters were measured and evaluated: total cholesterol (T-C), low-density lipoprotein cholesterol (LDL-C), high-density lipoprotein cholesterol (HDL-C), triacylglycerols (TG), and the inflammatory marker high-sensitivity C-reactive protein (hs-CRP).

The reference values for blood tests are as follows: T-C (total cholesterol) < 5.00 mmol/L, LDL-C (LDL cholesterol) 1.20–3.00 mmol/L, HDL-C (HDL cholesterol) 1.20–2.70 mmol/L, TG (triacylglycerols) 0.45–2.70 mmol/L, and the inflammatory marker hs-CRP (high-sensitive C-reactive protein) < 5.00 mg/L.

#### 2.3.2. Body Composition

Body composition parameters were determined using the InBody 720 device (Biospace Co., Ltd., Seoul, Republic of Korea). The InBody 720 device employs bioelectrical impedance analysis to determine several parameters, including fat mass (FM), fat-free mass (FFM), visceral fat area (VFA), percent body fat (PBF), skeletal muscle mass (SMM), total body water (TBW), intracellular water (ICW), and extracellular water (ECW). These parameters provide insights into the composition of the body and its individual segments. Additionally, the InBody 720 device measures body circumferences and calculates various indices such as body mass index (BMI), waist/hip ratio (WHR), and arm muscle circumference (AMC), etc.

Measurements should be taken on an empty stomach or at least 2 h after a meal and after using the toilet. It is recommended to avoid drinking a large volume of fluids, engaging in intense physical activity, taking a shower, or using a sauna before the examination, as these activities can cause temporary changes in body composition. During the body composition analysis, the room temperature should be maintained at 20–25 °C. The measurement results were processed using Lookin’Body 3.0 software (Biospace Co., Ltd., Seoul, Republic of Korea).

To determine the risk of cardiovascular disease (CVD) in menopausal women, we evaluated selected anthropometric parameters: visceral fat area (VFA, cm^2^), percentage of body fat (PBF, %), and body mass index (BMI, kg/m^2^). 

The body height of the subjects was measured using the TANITA WB-380H device (TANITA Co., Ltd., Tokyo, Japan), which measures both body height and weight simultaneously. During measurement, the subject stands upright with legs together and arms at the sides. The head is positioned in the Frankfurt plane. The distance from the top of the head (vertex) to the standing surface is measured. Subjects were measured without shoes and dressed only in underwear.

### 2.4. Statistical Evaluation of Results

The normality of values was assessed using the Shapiro–Wilk test. Differences between biochemical and anthropometric parameters with a normal distribution were tested by one-factor analysis of variance (ANOVA) and compared using Tukey’s post hoc test. Data are presented as mean values ± standard deviation (SD). For biochemical and anthropometric results with a non-normal distribution, the Wilcoxon test was used. These data are presented as median and interquartile range (IQR). 

For statistical processing of the obtained data, we used the Microsoft Office Excel 2010 program (Microsoft Corporation Co., Ltd., Los Angeles, CA, USA) in combination with XLSTAT (Version 2019.3.1). Statistical analyses were also performed using the program STATISTIKA Cz Version 10 (TIBCO Software Inc., Palo Alto, CA, USA). Statistical significance levels were set at *p* ˂ 0.05, *p* ˂ 0.01, and *p* ˂ 0.001.

Spearman’s rank correlation coefficient was used to summarize the strength and direction of the relationship between variables. Correlations were assessed using MedCalc^®^ Statistical Software version 22.021 (MedCalc Software Ltd., Ostend, Belgium; https://www.medcalc.org; accessed on 1 March 2024).

### 2.5. Ethical Considerations

The study was conducted in accordance with the Declaration of Helsinki and approved by the Ethics Committee at the Specialized Hospital of St. Zoerardus Zobor, n.o., Nitra, Kláštorská 131, 94901 Nitra, Slovakia (protocol number: 5/071220/2020; date of approval: 10 December 2020). The research complies with all applicable standards regarding research ethics and integrity.

## 3. Results

We evaluated a group of fifty-one menopausal women with a mean age of 47.49 ± 4.65 years (age range: 45–55 years). Their baseline characteristics are presented in Table 2. Typically, the basal metabolic rate (BMR) for women averages around 1400 kcal. The average BMR of the subjects was 1396.91 ± 112.34 kcal.

Based on the average waist/hip ratio (WHR) values (0.93 ± 0.07), we concluded that the subjects had WHR values indicative of central obesity. WHR values above 0.88 in women represent an increased risk of cardiovascular disease (CVD). The observed menopausal women reached approximately this value, as shown in Table 2.

### 3.1. Assessment of Lipid Profile in Relation to Flaxseed Intervention

In the lipid profile, we noted significant changes in T-C, LDL-C, and HDL-C levels in both groups of menopausal women who consumed flaxseed. The changes in individual lipoproteins were as follows.

Considering the variety of flaxseed, we observed a slight increase in T-C values (i.e., from 5.36 ± 0.89 mmol/L to 6.03 ± 0.83 mmol/L) in the high-lignan variety (HL; Agram) (*p* ˂ 0.05). In contrast, women consuming flaxseeds with a lower lignan content (LL; Raciol) experienced a more pronounced rise in T-C levels, from 5.66 ± 1.10 mmol/L to 6.40 ± 1.32 mmol/L, and these changes were highly significant (*p* < 0.001). The changes in the lipid profiles of menopausal women are presented in Table 3. 

Despite a slight increase in T-C, we noted a significant decrease in LDL-C particles. Consumption of the high-lignan variety (HL) adjusted the LDL-C level (*p* ˂ 0.001) to physiological values. Menopausal women consuming the flaxseed variety with a lower lignan content (LL; Raciol) also achieved LDL-C normalization (*p* ˂ 0.01).

We consider a significant increase in HDL-C levels to be another positive effect of the consumption of the monitored varieties of flaxseed. A better and more significant effect (*p* ˂ 0.001) was achieved by women in the group consuming the high-lignan variety (HL; Agram).

TG levels increased minimally as a result of the flaxseed intervention. They remained within the range of physiological values and can be considered almost stable. These changes were statistically insignificant.

Overall, we can conclude that the lipid profile of menopausal women was positively modulated. For a more significant benefit of this modulation (increase in HDL-C, decrease in LDL-C, and stable TG), we recommend consumption of flaxseed varieties with a high content of lignans.

### 3.2. Assessment of Another Cardiovascular Risk Indicator in Relation to Flaxseed Intervention

Another indicator that we can use to assess cardiovascular risk is hs-CRP level. C-reactive protein (CRP) is considered the most sensitive reactant of the acute phase of inflammation. CRP concentrations rise very quickly during inflammatory processes.

Through biochemical blood analysis, we found that the hs-CRP values were slightly increased before the intervention (compared to the physiological range). As a result of the intervention, hs-CRP levels significantly decreased and normalized with both consumed flaxseed varieties (HL, Agram, *p* ˂ 0.01; LL, Raciol, *p* ˂ 0.01).

The changes in selected cardiovascular risk indicators in menopausal women are presented in Table 4.

The visceral fat area (VFA) remained stable in all observed groups of menopausal women. A minimum (non-significant) reduction in visceral fat area (VFA) of 0.67 cm^2^ was observed in the group of women consuming the high-lignan variety of flaxseed (HL). 

In contrast, the data indicated a minimal increase in VFA in the LL group, which consumed a low-lignan variety of flaxseed. The observed differences were so minimal that they did not represent a change in the strength of the CVD risk.

The results of measuring VFA both before and after the intervention (values exceeding 100 cm^2^) indicated the potential presence of cardiovascular and metabolic risk factors in the cohort of menopausal women under observation. The impact of flaxseed consumption on VFA was not substantiated.

A comparable stable trend of value development was also observed in the PBF and BMI. A healthy percentage of body fat (PBF) for women aged 40–59 should be in the range of 23–33%. The values exhibited by the menopausal women in our study were within the aforementioned norm. The BMI assessment indicated borderline values between the normal and overweight categories (Table 4).

### 3.3. Correlations between Flaxseed Varieties and Monitored Cardiovascular Risk Indicators

The strength of the relationships between variables was analyzed using Spearman’s correlation analysis. We first evaluated the correlations regardless of the flaxseed variety consumed (Table 5); then specifically for the high-lignan variety, Agram (Table 6); and finally for the low-lignan variety, Raciol (Table 7).

Statistically significant positive associations were observed between BMI and VFA (r = 0.921; *p* < 0.0001) and BMI and PBF (r = 0.923; *p* < 0.0001), as well as between VFA and PBF (r = 0.962; *p* < 0.0001). These parameters are related to weight and the presence of adipose tissue, confirming very high correlations based on the correlation coefficients. Additionally, a high correlation was found between BMI and TG (r = 0.705; *p* < 0.0001) and between T-C and LDL-C (r = 0.727; *p* < 0.0001) (Table 5).

A moderate positive correlation was detected between PBF and TG (r = 0.615; *p* ˂ 0.0001) and between VFA and TG (r = 0.571; *p* ˂ 0.001).

Weak correlations were found between hs-CRP and TG (r = 0.499; *p* ˂ 0.05), hs-CRP and BMI (r = 0.467; *p* ˂ 0.05), hs-CRP and PBF (r = 0.457; *p* ˂ 0.05), and hs-CRP and VFA (r = 0.454; *p* ˂ 0.05). Low negative correlations were observed between HDL-C and BMI, HDL-C and PBF, and HDL-C and VFA (Table 5).

The associations between the monitored variables in relation to the high-lignan flaxseed variety (Agram) are presented in Table 6.

The analysis pointed to a strong correlation between the anthropometric variables BMI and PBF (r = 0.912; *p* ˂ 0.0001) and BMI and VFA (r = 0.846; *p* ˂ 0.0001). These relationships were slightly weaker compared to the overall assessment regardless of the flaxseed variety or lignan content. 

We also noted a strong correlation between hs-CRP and BMI (r = 0.646; *p* ˂ 0.05), which was stronger compared to the overall assessment regardless of the lignan content (Table 5). Similarly, significant correlations were found between hs-CRP and VFA (r = 0.552; *p* ˂ 0.05), hs-CRP and PBF (r = 0.557; *p* ˂ 0.05), and T-C and LDL-C (r = 0.855; *p* ˂ 0.0001).

A moderate negative correlation was found between HDL-C and VFA (r = -0.521; *p* < 0.05).

The correlation analysis for the influence of the low-lignan flax variety (Raciol) revealed strong positive correlations between BMI and VFA (r = 0.944; *p* ˂ 0.0001), BMI and PBF (r = 0.912; *p* ˂ 0.0001), and PBF and VFA (r = 0.934; *p* ˂ 0.0001).

Moderate positive correlations were confirmed between T-C and TG (r = 0.697; *p* ˂ 0.05), BMI and TG (r = 0.693; *p* ˂ 0.05), PBF and TG (r = 0.680; *p* ˂ 0.05), and TG and VFA (r = 0.609; *p* ˂ 0.05). We also detected a moderate correlation between BMI and LDL-C, PBF and LDL-C, T-C and LDL-C, TG and LDL-C, and hs-CRP and TG.

Weak negative correlations were observed between HDL-C and BMI, HDL-C and PBF, HDL-C and LDL-C, and HDL-C and VFA. 

Table 7 shows the correlations of the monitored parameters for the low-lignan variety.

## 4. Discussion

Our study focused on analyzing the effects of consuming crushed flaxseeds from two different varieties on selected risk factors of cardiovascular diseases. The cohort consisted of menopausal women from the general population, without any diagnosis of serious diseases. Clinical research in several studies has confirmed that menopause is a critical period for the emergence and development of cardiovascular diseases [25,26,27] for several reasons.

Hormonal changes associated with menopause, particularly the decrease in endogenous estrogen production [28], contribute to the development of increases in fat mass, enlargement of the visceral fat area, changes in the lipid profile, and the development of hypertension due to disturbed lipid metabolism [28,29,30].

The average age of the menopausal women in our study was 47.49 ± 4.65 years, which falls within the typical menopause age range of 45 to 55 years. The onset of menopause before the age of 45 is considered early menopause [31]. Early menopause may be more detrimental to women’s cardiovascular health due to the early termination of estrogenic cardiovascular protection [27]. Early menopause has been associated with an increased risk of coronary heart disease [31,32,33], heart failure [34], and, to a lesser extent, stroke [33,35]. Considering these findings, the risk of cardiovascular diseases and their complications in the women in our study corresponds to the typical onset of menopause.

Common symptoms of menopause include vaginal dryness, hot flashes, night sweats, and bone pain. Traditionally, Linum usitatissimum (flaxseed) has been used to treat postmenopausal symptoms [36]. In our study, many women reported a reduction in or elimination of menopause symptoms after an 8-week intervention with crushed flaxseeds. Similarly, Cetisli et al. [37] observed that after 3 months of flaxseed consumption, postmenopausal women experienced a suppression of menopausal symptoms, thereby improving their quality of life.

Regarding the cardiovascular disease (CVD) risk associated with menopause, it primarily involves changes in the lipid profile during this transition. These changes typically include an increase in total cholesterol (T-C), low-density lipoprotein cholesterol (LDL-C), and triacylglycerols (TG) along with a decrease in high-density lipoprotein cholesterol (HDL-C) [38]. Flaxseed has been shown to reduce TG, T-C, LDL-C, and the T-C/HDL-C ratio in a dose-dependent manner [39]. In the menopausal women we observed, considering the variety of flaxseed consumed, we found a slight increase in T-C values (from 5.36 ± 0.89 mmol/L to 6.03 ± 0.83 mmol/L) in the high-lignan variety group (*p* ˂ 0.05). In those consuming flaxseeds with a lower lignan content, the increase in T-C levels was more pronounced (from 5.66 ± 1.10 mmol/L to 6.40 ± 1.32 mmol/L) (*p* ˂ 0.001). Despite this slight increase in T-C, we noted a significant decrease in LDL-C particles. Consumption of the high-lignan variety adjusted the LDL-C level from 3.26 ± 0.7 mmol/L to 2.65 ± 0.58 mmol/L (*p* ˂ 0.001), i.e., to physiological values. Similarly, menopausal women consuming the lower lignan content variety also achieved LDL-C normalization (*p* ˂ 0.01). We also observed minimally increased TG after the intervention of both flaxseed varieties (LL and HL). Despite this, TG levels remained within physiological range and were almost stable post-intervention. Regarding TG, we did not observe a significant reduction, possibly because the participants had normal TG levels initially, or a longer intervention period may be needed to achieve reduction. In our overall evaluation, we identified anti-dyslipidemic properties in the high-lignan variety of flaxseed. Similar findings were reported in the study by Parikh et al. [40].

The hs-CRP value can be used as an indicator of cardiovascular risk, specifically as a marker for predicting the risk of developing coronary heart disease in healthy individuals. Additionally, the hs-CRP value can be used to assess the prognosis of cardiovascular events given its close relationship to the “inflammatory” theory of the pathogenesis of atherosclerosis [41,42]. In our study, we initially observed slightly elevated hs-CRP levels before the intervention, indicating an increased cardiovascular risk compared to physiological norms. After the intervention, hs-CRP levels decreased significantly and normalized with both consumed flaxseed varieties. Basal CRP levels can be influenced by several factors, many of which are modifiable. Increased CRP levels are associated with factors such as unhealthy lifestyle (smoking, sedentary behavior, and higher BMI), metabolic disorders (obesity, low HDL-C, high LDL-C, elevated TG levels, hypertension, metabolic syndrome, and diabetes mellitus), chronic infections, inflammatory conditions, and the use of estrogen and progesterone therapies. Conversely, factors that reduce CRP levels include increased physical activity, weight loss, smoking cessation, moderate alcohol consumption, and certain medications such as statins [43,44]. Before the intervention, the menopausal women in our study exhibited high cardiovascular risk with elevated hs-CRP levels (>3.0 mg/L), averaging 5.37 ± 4.65 mg/L. Following the 8-week intervention with crushed flaxseeds, there was a significant reduction in hs-CRP levels, leading to a gradual mitigation of cardiovascular disease risk. Post-intervention, the female subjects reached an intermediate risk category for CVD (hs-CRP: 1–3 mg/L). However, achieving hs-CRP values indicative of low cardiovascular risk (<1 mg/L) may require a longer intervention period. The association between hs-CRP levels and CVD risk has been corroborated by other studies as well [45,46].

Anthropometric values are closely related to genetic factors, environmental conditions, social and cultural factors, lifestyle choices, functional status, and overall health. Anthropometric measurements can serve to assess the risk of malnutrition, obesity, muscle wasting, increased fat mass, maldistribution of adipose tissue, and cardiovascular risk [47,48,49].

The visceral fat area (VFA) remained consistent across all groups of menopausal women observed in our study. However, the initial and post-intervention measurements of VFA (values exceeding 100 cm^2^) indicated an elevated cardiovascular and metabolic risk among the women studied. This aligns with findings from Zając-Gawlak et al., who also identified high VFA levels as a significant risk factor for cardiovascular disease and metabolic syndrome in menopausal women [50]. Despite our study, the effect of flaxseed consumption on reducing VFA was not confirmed.

Similarly, stable trends in value development were observed in PBF and BMI. According to established norms, a healthy percentage of body fat (PBF) for women aged 40–59 should range between 23% and 33%. Our menopausal women exhibited a PBF of 32.01 ± 8.22%, indicating that they fall within this healthy range. BMI assessments revealed values that border on the threshold between normal weight and overweight, at approximately 26 kg/m^2^.

Our finding that the flaxseed variety with a higher lignan content (6248 mg/kgSECO; brown seed color) had a greater effect on WFA, PBF, and BMI than the variety with a lower lignan content (4297.2 mg/kgSECO; yellow seed color) contradicts the study by Aguilar et al., who found that brown flaxseed was less effective than golden flaxseed in modifying body composition in perimenopausal women [51]. For the high-lignan flaxseed variety, we observed a strong correlation between hs-CRP and BMI (r = 0.646; *p* ˂ 0.05), hs-CRP and VFA (r = 0.552; *p* ˂ 0.05), and hs-CRP and PBF (r = 0.557; *p* ˂ 0.05). Additionally, there was a very strong correlation between T-C and LDL-C (r = 0.855; *p* ˂ 0.0001). A moderate negative correlation was noted between HDL-C and VFA (r = −0.521; *p* ˂ 0.05).

In contrast, the correlation analysis for the low-lignan flax variety revealed moderate positive correlations between T-C and TG (r = 0.697; *p* ˂ 0.05), BMI and TG (r = 0.693; *p* ˂ 0.05), PBF and TG (r = 0.680; *p* ˂ 0.05), and TG and VFA (r = 0.609; *p* ˂ 0.05). Additionally, we detected moderate correlations between BMI and LDL-C, PBF and LDL-C, T-C and LDL-C, TG and LDL-C, and hs-CRP and TG.

Using whole flaxseed at doses of 30 g/day or more over longer-term interventions (12 weeks or more) in participants with a higher BMI (27 kg/m^2^ or more) has shown positive effects on body composition. Whole flaxseed is particularly effective for weight management, especially in reducing weight in overweight and obese participants [52]. In our study, we did not observe significant changes in body composition, which may be due to the shorter duration of the intervention (8 weeks) and the dose of 40 g/day of crushed flaxseeds. The menopausal women in our study had borderline values of BMI between normal and overweight (approximately 26 kg/m^2^), VFA (approximately 102 cm^2^), and PBF (approximately 32%).

### Study Limitations

Although our sample size is consistent with similar published studies, increasing the sample size in future research would be beneficial. Additional data could enhance the consistency and reliability of our findings. A potential limitation of our study is the difference in fatty acid intake between participants consuming flaxseed and those in the control group who did not consume flaxseed. In future studies, it would be advisable to use a placebo for the control group.

## 5. Conclusions

Our study focused on monitoring the effect of crushed flaxseeds from two varieties with differing lignan contents on cardiovascular disease risk factors. We found that an 8-week intervention with crushed flaxseeds already led to significant improvements and normalization of several cardiovascular risk factors, including LDL-C, HDL-C, and hs-CRP. The variety with a higher lignan content demonstrated a stronger effect, which was also confirmed by the correlation analysis. Specifically, we observed a decrease in LDL-C and hs-CRP and an increase in HDL-C, which are beneficial for the cardiovascular system and health. However, a slight increase in T-C and TG may pose a concern for people/patients with already elevated blood levels. The intervention did not significantly affect BMI, PBF, or VFA parameters, which remained relatively stable throughout the study. Therefore, the modulation of CVD risk factors depends on the daily dose, duration, form of consumption, and lignan content of the flaxseed variety.

## Figures and Tables

**Table 1:** Selected nutritional parameters of flaxseed varieties.

Variety of Flax	Lignans—SECO (mg/kg)	Linoleic Acid (%)	α-Linolenic Acid (%)	Oleic Acid (%)	PUFA (%)	MUFA (%)	SFA (%)	Fiber g/kg
Agram (CZ ^1^)	6248.0	27.6	41.6	18.7	41.6	46.5	10.6	86.3
Raciol (CZ ^1^)	4297.2	39.4	34.535	14.9	34.5	54.4	10.0	153.2

^1^ The origin of the flax; SECO—secoisolariciresinol; PUFA—polyunsaturated fatty acid; MUFA—monounsaturated fatty acid; SFA—saturated fatty acid.

**Table 2:** Baseline characteristics of menopausal women.

Parameter	All (*n* = 51) Mean ± SD	HL (*n* = 17) Mean ± SD	LL (*n* = 17) Mean ± SD	C (*n* = 17) Mean ± SD
Age (years)	47.49 ± 4.65	49.76 ± 4.76	48.18 ± 3.30	45.49 ± 3.25
Height (cm)	168.31 ± 5.68	169.94 ± 3.90	166.24 ± 5.25	168.74 ± 4.50
Weight (kg)	71.68 ± 13.84	75.3 ± 15.78	72.63 ± 14.49	67.09 ± 10.12
Waist/hip ratio	0.93 ± 0.07	0.94 ± 0.07	0.94 ± 0.07	0.91 ± 0.05
Body mass index (kg/m^2^)	25.49 ± 4.93	26.15 ± 5.87	26.19 ± 5.32	24.11 ± 3.20
Basal metabolic rate (kcal)	1396.91 ± 112.34	1449.07 ± 92.20	1388.36 ± 104.42	1353.30 ± 122.65
Minimum caloric need (kcal)	1459.69 ± 302	1497.50 ± 236.14	1451.89 ± 221.12	1429.67 ± 248.16
Maximum caloric need (kcal)	1730.01 ± 291	1748.06 ± 288.62	1692.31 ± 270.26	1749.67 ± 275.87
Body condition status (point)	70.73 ± 7.54	71.06 ± 7.98	69.65 ± 7.91	71.47 ± 7.02

HL—high-lignan group (variety Agram); LL—low-lignan group (variety Raciol); C—control group. Data are presented as mean ± SD before intervention.

**Table 3:** Changes in lipid profiles of menopausal women.

Parameter		All (*n* = 51) Mean ± SD	HL (*n* = 17) Mean ± SD	LL (*n* = 17) Mean ± SD	C (*n* = 17) Mean ± SD
T-C (mmol/L)	before	5.79 (1.43)	5.36 ± 0.89	5.66 ± 1.10	6.80 ± 1.32
	after	6.27 (1.31)	6.03 ± 0.83	6.40 ± 1.32	6.77 ± 1.46
	*p*	0.0016	0.0137	0.0001	0.8947
LDL-C (mmol/L)	before	3.43 (1.39)	2.89 (1.19)	3.43 ± 0.87	4.31 ± 1.32
	after	2.94 (1.13)	2.44 (0.67)	2.80 ± 0.85	3.88 ± 1.32
	*p*	0.0000	0.0004	0.0023	0.0275
HDL-C (mmol/L)	before	1.70 ± 0.36	1.75 ± 0.39	1.71 ± 0.35	1.73 ± 0.38
	after	1.91 ± 0.45	1.99 ± 0.41	2.04 ± 0.49	1.82 ± 0.42
	*p*	0.0000	0.0000	0.0017	0.0671
TG (mmol/L)	before	1.06 (0.66)	0.99 (0.86)	0.9 (0.71)	1.26 (0.36)
	after	0.95 (0.61)	1.08 (0.81)	0.95 (0.79)	0.92 (0.84)
	*p*	0.4572	0.6874	0.4691	0.0157

HL—high-lignan group (variety Agram); LL—low-lignan group (variety Raciol); C—control group; T-C—total cholesterol; LDL-C—LDL cholesterol; HDL-C—HDL cholesterol; TG—triacylglycerols; *p*—*p*-value. Data are presented as mean ± SD for normally distributed values or median (IQR) for not normally distributed values.

**Table 4:** Changes in selected cardiovascular risk indicators in menopausal women.

Parameter		All (*n* = 51) Mean ± SD	HL (*n* = 17) Mean ± SD	LL (*n* = 17) Mean ± SD	C (*n* = 17) Mean ± SD
hs-CRP (mg/L)	Before	4.39 (4.98)	5.15 (4.66)	5.09 (3.93)	1.32 (4.64)
	After	1.86 (3.92)	2.71 (4.38)	1.86 (3.54)	1.57 (3.21)
	*p*	0.0000	0.0019	0.0011	0.8313
VFA (cm^2^)	Before	97.27 ± 36.18	102.62 ± 41.30	82.37 (22.75)	88.10 ± 26.61
	After	97.48 ± 36.45	101.95 ± 40.98	83.19 (15.98)	88.17 ± 26.61
	*p*	0.6959	0.4924	0.0928	0.9285
PBF (%)	Before	32.31 ± 8.25	32.12 ± 8.74	33.24 ± 9.27	31.57 ± 6.97
	After	32.01 ± 8.22	31.71 ± 8.44	33.06 ± 9.57	31.28 ± 6.80
	*p*	0.0555	0.1946	0.4614	0.2460
BMI (kg/m^2^)	Before	24.2 (5.04)	26.15 ± 5.87	22.84 (2.65)	24.11 ± 3.20
	After	24.42 (4.99)	26.08 ± 5.74	23.01	24.10 ± 3.24
	*p*	0.8374	0.3210	0.2559	0.8901

HL—high-lignan group (variety Agram); LL—low-lignan group (variety Raciol); C—control group; hs-CRP—high-sensitivity CRP; VFA—visceral fat area; PBF—percentage of body fat; BMI—body mass index; *p*—*p*-value. Data are presented as mean ± SD for normally distributed values or median (IQR) for not normally distributed values.

**Table 5:** Correlations for the monitored parameters regardless of the flaxseed variety.

	Varieties	BMI	VFA	PBF	T-C	LDL-C	HDL-C	TG	hs-CRP
**Varieties**	1	0.06	0.045	0.093	0.159	0.114	−0.003	−0.153	0.033
		*p* = 0.7362	*p* = 0.8006	*p* = 0.6011	*p* = 0.3694	*p* = 0.5211	*p* = 0.9866	*p* = 0.3878	*p* = 0.8531
**BMI**	0.06	1	0.921	0.923	0.379	0.436	−0.449	0.705	0.467
	*p* = 0.7362		*p* < 0.0001	*p* < 0.0001	*p* = 0.0269	*p* = 0.0099	*p* = 0.0077	*p* < 0.0001	*p* = 0.0053
**VFA**	0.045	0.921	1	0.962	0.274	0.339	−0.426	0.571	0.454
	*p* = 0.8006	*p* < 0.0001		*p* < 0.0001	*p* = 0.1162	*p* = 0.0502	*p* = 0.0121	*p* = 0.0004	*p* = 0.0071
**PBF**	0.093	0.923	0.962	1	034	0.413	−0.461	0.615	0.457
	*p* = 0.6011	*p* < 0.0001	*p* < 0.0001		*p* = 0.0495	*p* = 0.0153	*p* = 0.0060	*p* = 0.0001	*p* = 0.0065
**T-C**	0.159	0.379	0.274	0.34	1	0.727	0.303	0.44	0.093
	*p* = 0.3694	*p* = 0.0269	*p* = 0.1162	*p* = 0.0495		*p* < 0.0001	*p* = 0.0811	*p* = 0.0092	*p* = 0.6024
**LDL-C**	0.114	0.436	0.339	0.413	0.727	1	−0.181	0.352	−0.007
	*p* = 0.5211	*p* = 0.0099	*p* = 0.0502	*p* = 0.0153	*p* < 0.0001		*p* = 0.3053	*p* = 0.0412	*p* = 0.9706
**HDL-C**	−0.003	−0.449	−0.426	−0.461	0.303	−0.181	1	−0.258	−0.27
	*p* = 0.9866	*p* = 0.0077	*p* = 0.0121	*p* = 0.0060	*p* = 0.0811	*p* = 0.3053		*p* = 0.1410	*p* = 0.1220
**TG**	−0.153	0.705	0.571	0.615	0.44	0.352	−0.258	1	0.499
	*p* = 0.3878	*p* < 0.0001	*p* = 0.0004	*p* = 0.0001	*p* = 0.0092	*p* = 0.0412	*p* = 0.1410		*p* = 0.0027
**hs-CRP**	0.033	0.467	0.454	0.457	0.093	−0.007	−0.27	0.499	1
	*p* = 0.8531	*p* = 0.0053	*p* = 0.0071	*p* = 0.0065	*p* = 0.6024	*p* = 0.9706	*p* = 0.1220	*p* = 0.0027	

BMI—body mass index; VFA—visceral fat area; PBF—percentage of body fat; T-C—total cholesterol; LDL-C—LDL cholesterol; HDL-C—HDL cholesterol; TG—triacylglycerols; hs-CRP—high-sensitivity CRP. Data are presented as correlation coefficient r/*p*-value.

**Table 6:** Correlations of the monitored parameters for the variety Agram (HL).

HL (Agram)	BMI	VFA	PBF	T-C	LDL-C	HDL-C	TG	hs-CRP
**BMI**	1	0.846	0.912	0.275	0.353	−0.455	0.79	0.646
		*p* < 0.0001	*p* < 0.0001	*p* = 0.2863	*p* = 0.1641	*p* = 0.0665	*p* = 0.0002	*p* = 0.0051
**VFA**	0.846	1	0.924	0.064	0.189	−0.521	0.482	0.552
	*p* < 0.0001		*p* < 0.0001	*p* = 0.8080	*p* = 0.4676	*p* = 0.0319	*p* = 0.0501	*p* = 0.0216
**PBF**	0.912	0.924	1	0.265	0.373	−0.465	0.569	0.557
	*p* < 0.0001	*p* < 0.0001		*p* = 0.3045	*p* = 0.1403	*p* = 0.0602	*p* = 0.0171	*p* = 0.0203
**T-C**	0.275	0.064	0.265	1	0.855	0.341	0.202	−0.022
	*p* = 0.2863	*p* = 0.8080	*p* = 0.3045		*p* < 0.0001	*p* = 0.1806	*p* = 0.4361	*p* = 0.9330
**LDL-C**	0.353	0.189	0.373	0.855	1	0.077	0.231	−0.13
	*p* = 0.1641	*p* = 0.4676	*p* = 0.1403	*p* < 0.0001		*p* = 0.7697	*p* = 0.3728	*p* = 0.6186
**HDL-C**	−0.455	−0.521	−0.465	0.341	0.077	1	−0.402	−0.369
	*p* = 0.0665	*p* = 0.0319	*p* = 0.0602	*p* = 0.1806	*p* = 0.7697		*p* = 0.1093	*p* = 0.1446
**TG**	0.79	0.482	0.569	0.202	0.231	−0.402	1	0.453
	*p* = 0.0002	*p* = 0.0501	*p* = 0.0171	*p* = 0.4361	*p* = 0.3728	*p* = 0.1093		*p* = 0.0680
**hs-CRP**	0.646	0.552	0.557	−0.022	−0.13	−0.369	0.453	1
	*p* = 0.0051	*p* = 0.0216	*p* = 0.0203	*p* = 0.9330	*p* = 0.6186	*p* = 0.1446	*p* = 0.0680	

BMI—body mass index; VFA—visceral fat area; PBF—percentage of body fat; T-C—total cholesterol; LDL-C—LDL cholesterol; HDL-C—HDL cholesterol; TG—triacylglycerols; hs-CRP—high-sensitivity CRP. Data are presented as correlation coefficient r/*p*-value.

**Table 7:** Correlations of the monitored parameters for the variety Raciol (LL).

LL (Raciol)	BMI	VFA	PBF	T-C	LDL-C	HDL-C	TG	hs-CRP
**BMI**	1	0.944	0.912	0.434	0.549	−0.422	0.693	0.324
		*p* < 0.0001	*p* < 0.0001	*p* = 0.0819	*p* = 0.0225	*p* = 0.0919	*p* = 0.0020	*p* = 0.2052
**VFA**	0.944	1	0.934	0.331	0.456	−0.373	0.609	0.311
	*p* < 0.0001		*p* < 0.0001	*p* = 0.1945	*p* = 0.0659	*p* = 0.1408	*p* = 0.0095	*p* = 0.2239
**PBF**	0.912	0.934	1	0.382	0.551	−0.426	0.68	0.338
	*p* < 0.0001	*p* < 0.0001		*p* = 0.1299	*p* = 0.0217	*p* = 0.0878	*p* = 0.0027	*p* = 0.1842
**T-C**	0.434	0.331	0.382	1	0.522	0.304	0.697	0.154
	*p* = 0.0819	*p* = 0.1945	*p* = 0.1299		*p* = 0.0316	*p* = 0.2356	*p* = 0.0019	*p* = 0.5540
**LDL-C**	0.549	0.456	0.551	0.522	1	−0.419	0.542	0.091
	*p* = 0.0225	*p* = 0.0659	*p* = 0.0217	*p* = 0.0316		*p* = 0.0940	*p* = 0.0245	*p* = 0.7292
**HDL-C**	−0.422	−0.373	−0.426	0.304	−0.419	1	−0.212	−0.162
	*p* = 0.0919	*p* = 0.1408	*p* = 0.0878	*p* = 0.2356	*p* = 0.0940		*p* = 0.4134	*p* = 0.5351
**TG**	0.693	0.609	0.68	0.697	0.542	−0.212	1	0.539
	*p* = 0.0020	*p* = 0.0095	*p* = 0.0027	*p* = 0.0019	*p* = 0.0245	*p* = 0.4134		*p* = 0.0257
**hs-CRP**	0.324	0.311	0.338	0.154	0.091	−0.162	0.539	1
	*p* = 0.2052	*p* = 0.2239	*p* = 0.1842	*p* = 0.5540	*p* = 0.7292	*p* = 0.5351	*p* = 0.0257	

BMI—body mass index; VFA—visceral fat area; PBF—percentage of body fat; T-C—total cholesterol; LDL-C—LDL cholesterol; HDL-C—HDL cholesterol; TG—triacylglycerols; hs-CRP—high-sensitivity CRP. Data are presented as correlation coefficient r/*p*-value.

## Data Availability

The original contributions presented in the study are included in the article, further inquiries can be directed to the corresponding author.
